# Fibroblast Growth Factor in Diabetic Foot Ulcer: Progress and Therapeutic Prospects

**DOI:** 10.3389/fendo.2021.744868

**Published:** 2021-10-14

**Authors:** Ye Liu, Yiqiu Liu, Junyu Deng, Wei Li, Xuqiang Nie

**Affiliations:** ^1^ College of Pharmacy, Zunyi Medical University, Zunyi, China; ^2^ Joint International Research Laboratory of Ethnomedicine of Chinese Ministry of Education, Zunyi Medical University, Zunyi, China; ^3^ Key Lab of the Basic Pharmacology of the Ministry of Education, Zunyi Medical University, Zunyi, China

**Keywords:** fibroblast growth factor, diabetic foot ulcers, signaling pathways, wound healing, mechanism

## Abstract

Diabetic foot ulcer (DFU) is a combination of neuropathy and various degrees of peripheral vasculopathy in diabetic patients resulting in lower extremity infection, ulcer formation, and deep-tissue necrosis. The difficulty of wound healing in diabetic patients is caused by a high glucose environment and various biological factors in the patient. The patients’ skin local microenvironment changes and immune chemotactic response dysfunction. Wounds are easy to be damaged and ulcerated repeatedly, but difficult to heal, and eventually develop into chronic ulcers. DFU is a complex biological process in which many cells interact with each other. A variety of growth factors released from wounds are necessary for coordination and promotion of healing. Fibroblast growth factor (FGF) is a family of cell signaling proteins, which can mediate various processes such as angiogenesis, wound healing, metabolic regulation and embryonic development through its specific receptors. FGF can stimulate angiogenesis and proliferation of fibroblasts, and it is a powerful angiogenesis factor. Twenty-three subtypes have been identified and divided into seven subfamilies. Traditional treatments for DFU can only remove necrotic tissue, delay disease progression, and have a limited ability to repair wounds. In recent years, with the increasing understanding of the function of FGF, more and more researchers have been applying FGF-1, FGF-2, FGF-4, FGF-7, FGF-21 and FGF-23 topically to DFU with good therapeutic effects. This review elaborates on the recently developed FGF family members, outlining their mechanisms of action, and describing their potential therapeutics in DFU.

## Introduction

Diabetes is a major health issue that has reached alarming levels. The prevalence of diabetes has been increasing worldwide for approximately 50 years and has reached epidemic proportions globally and in China. It is predicted to rise to 10.2% (578 million) by 2030 and 10.9% (700 million) by 2045 (1), which means that one in ten people have diabetes. Diabetes and its complications have a significantly economic impact on individuals, families, national economy, and global health system. Health spending on diabetes is expected to grow to $825 billion by 2030 and to $845 billion by 2045 (2). The chronic complications of diabetes vary with the type of diabetes, onset time and metabolic control degree, and the most common complications are neuropathy (42.1%), retinopathy (44%), nephropathy (63.1%) and macroangiopathy (43%) (3).

Peripheral neuropathy and peripheral vascular disease of the lower limbs can occur in diabetic patients due to their constant exposure to high glucose (4), but such neuropathy and vascular disease are often not easily detected. If a wound develops in the lower limb of a diabetic patient at this time, it can become highly susceptible to infection, which can lead to foot ulcers and, if not appropriately treated, can progress to partial or complete amputation of the lower limb. It is known that about one in six diabetic patients worldwide will have at least one plantar ulcer in their lifetime. The probability of foot ulcers in patients with diabetes 3 – 5 years is estimated to be 2.4 – 2.6%, with a prevalence of 4 – 10%. In addition, diabetic foot ulcers are prone to relapse, and the risk of developing DFU is about 2% per year in most diabetics, but for patients with a history of DFU, the risk of recurring DFU will increase to 17% -60% in the next three years (5). Generally speaking, the healing process of a typical wound includes four stages: hemostasis, inflammation, proliferation, and remodeling. However, the environment of continuous hyperglycemia in diabetic patients affects various processes of routine wound healing. It is reported that diabetic patients will have hypercoagulable state and skin function decline during hemostasis (6). During the process of inflammation, the imbalance of some inflammatory factors and several growth factors in diabetic patients leads to the long-term chronic inflammatory reaction of wounds (7), and it is reported that the reduction of neutrophil function is also one of the reasons leading to the susceptibility of diabetic wounds (8). Due to long-term exposure to high sugar, the migration and proliferation of keratinocytes in diabetic patients decreased, resulting in insufficient wound re-epithelialization, which further affected the wound healing process (9). The differential expression of extracellular matrix produced, assembled and remodeled by fibroblasts also leads to poor healing of diabetic wounds (10). DFU not only leads to high medical expenses and overcrowding in clinics, but also often causes ulcers. Wounds that are hard to heal bring great physical and mental pain and torture to patients. As the population ages and the incidence of diabetes increases, so does the prevalence of DFU in young people (11). According to studies, the older you are and the longer you have diabetes, the more likely you are to develop DFU (12). Therefore, treating DFU is one of the most critical health problems that urgently need to be addressed in clinical practice.

In recent years, with the in-depth study of wound healing process, it has been found that many growth factors are closely related to the repair cells, especially play a key role of wound repair, among which fibroblast growth factor (FGF) is one of them (13). Fibroblast growth factor (FGF) is a kind of polypeptide growth factors with various biological activities, which widely exists in various organs and tissues. Twenty-three FGF family members have been found, which are divided into 7 subfamilies (14). Secretory fibroblast growth factor is expressed in almost all tissues and plays an important role in the early stages of embryo development. In adulthood, it acts as a balance factor in the body and is important for tissue maintenance, repair, regeneration and metabolism (14). Secreted FGFs fall into two categories, namely classical FGFs (also known as paracrine FGFs) and endocrine FGFs. In addition to the typical FGF function of controlling cell proliferation, differentiation and survival, endocrine FGF also regulates the metabolism of phosphate, bile acid, carbohydrate and lipid. Paracrine FGFs functions include neural development, keratinocyte organization, angiogenesis, and wound healing processes, and any functional abnormality leads to a range of developmental defects. Fibroblast growth factors is a stronger angiogenesis factors than platelet-derived growth factor (PDGF) and vascular endothelial growth factor (VEGF). FGFs stimulates angiogenesis and proliferation of fibroblasts, forming granulation tissue. In the initial stages of the wound healing process, tissue fills the space of the wound and the cavity of the wound (15).

Many reports suggest that some FGF subtypes may affect the healing process of diabetic wounds, such as aFGF, bFGF and FGF 15/19 subfamily, which has become a research hotspot. It has been found that aFGF significantly increases the number of capillaries and fibroblasts in ulcer tissue, and enhances the expression of TGF-í and PCNA proliferative proteins, thus promoting the healing of diabetic ulcer (16). Another study showed that in a diabetic mouse model, by controlling the release of bFGF, the healing of skin wound was accelerated, and the rate of epithelial formation increased. In addition, controlled release of bFGF can induce apoptosis of fibroblasts and myofibroblasts in the wound area, thus reducing scar formation during healing (17). Earlier reports have found that FGF-19 and FGF-21 are abnormally expressed in the serum of diabetic patients (18, 19), indicating that they regulate major metabolic processes in an endocrine way, including metabolism of blood sugar, blood lipids, cholesterol and bile acid. The articles related to FGFs and DFU were identified by searching major relevant literature databases including PubMed, Elsevier, China National Knowledge Infrastructure (CNKI), Chinese VIP Information (VIP), EMBase, Cochrane Library, Web of Science, and Wanfang, up to May 2021. The primary objective of this review was to investigate the possible mechanisms underlying FGF subtypes and recombinant FGF related to DFU, and to identify potential therapeutic targets.

## DFU Pathophysiological Mechanism

The skin barrier defects that has not healed within 3 months, that is, chronic wound, have become the main treatment challenge nowadays, and is increasingly associated with an aging population and the incidence of diabetes, obesity and vascular disease. Healing of damaged skin involves complex and interlocking interactions between many cytokines in the skin barrier (20). Wound healing usually goes through the following stages: hemostasis, inflammation, proliferation, contraction and remodeling. However, clinical and experimental studies have found that the healing process of diabetic wounds does not strictly follow the above-mentioned normal stages of wound healing, and stagnates at different stages, leading to ulcers and delaying wound healing.

According to previous studies, the healing of diabetic wounds can be affected by the following factors: 1. Excessive oxidative stress: it is in a state of high glucose continuously, and excessive redox and products affect all stages of wound healing, and inhibit its healing. At the wound, the generation and removal of reactive oxygen species (ROS) is necessary to ensure wound healing, while diabetic wounds present high levels of reactive oxygen species. High glucose can lead to an increase of substrates for energy metabolism, thus producing excessive superoxide, and promoting the increase in oxidative stress and its corresponding products (21). These products further induce the generation of advanced glycation end products (22). Decoupling of nitric oxide synthase leads to the decrease of nitric oxide production (23), which can make wound healing difficult.

**Figure 1:**
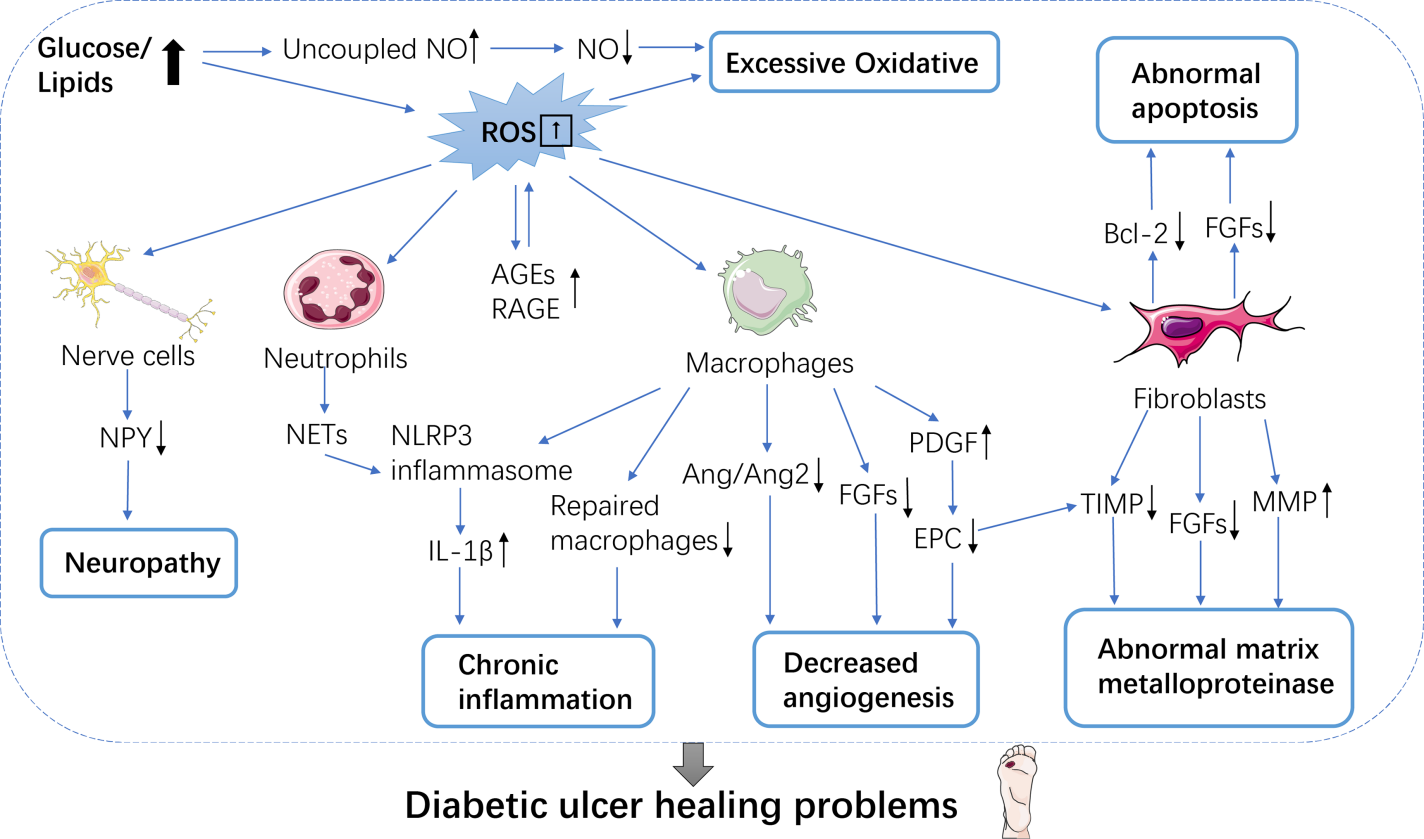
Mechanisms of diabetic wound healing difficulties. Wound healing is a complex array of multiple processes, many of which are mediated by growth factors. Six mechanisms make it difficult for diabetic patients to heal wounds. These include excessive oxidative; neuropathy; chronic inflammation; decreased angiogenesis; abnormal matrix metalloproteinase and abnormal apoptosis. NO, Nitric oxide; ROS, Reactive oxygen species; AGEs/RAGE, Advanced glycosylated end-products/glycosylated end product receptor; NPY, Neuropeptide Y; NETs, Extracellular traps; IL-1β, Interleukin-1β; Ang1/Ang2, Angiogenin 1/angiogenin 2; PDGF, Platelet-derived growth factor; EPCs, endothelial progenitor cells; TIMP, Metalloproteinase inhibitor; MMPs, Matrix metalloproteinases; Bcl-2, B cell lymphoma-2.

2. Excessive inflammatory reaction: In the inflammatory stage of a normal wounds, neutrophils and monocytes migrate to the wound site and release various cytokines and growth factors. Recent studies have demonstrated that that the diabetic group has a higher proportion of T cells and more inflammatory cell clusters such as NK cells, B cells, and mast cells. In contrast, the DFU group has a higher proportion of endothelial cells and smooth muscle cells (24). However, it is difficult to transition the inflammatory stage to the proliferative stage of the wound in diabetic patients, resulting in a long-term chronic inflammatory state in patients (25). Studies have found that excessive chronic inflammation to acute inflammation levels in DFU wounds is the key to wound healing (26, 27). In fact, a more recent study suggests that inhibition of FOXM1 leads to an acute to chronic healing deficit phenotype switch and delays wound healing., In DFU, FOXM 1 and the interactive proteins promoting wound healing were down-regulated, indicating that the FOXM 1 pathway was inhibited by pathology (28). When the inflammatory phase of injured tissues in diabetic patients is in the late stage, macrophages are still in an inflammatory state, which cannot be transformed into a repair phenotype, and then cannot secrete the medium to promote tissue repair, and the wound cannot transition to the proliferative phase, leading to chronic inflammation (29). It is also found that excessive neutrophils release extracellular traps (NETs) in diabetic wounds, which activate NLRP 3 inflammasome in macrophages and release IL-1β, and inflammatory cytokines exist in diabetic wounds for much longer time than normal, which may also be the reason for prolonging the inflammatory period and inhibiting granulation tissue formation (30).

3. Decreased angiogenesis: Insufficient angiogenesis in the wounds of diabetic patients mainly affects the proliferative stage of the wounds. In the proliferative phase of wound healing in normal subjects, the number of wound blood vessels is much higher than that in normal skin. It was first shown that in wounds, macrophages are a major source of vascular endothelial growth factor (VEGF) and other pro-angiogenic factors (31). However, during the inflammatory stage of the wound, the transformation of macrophages into repair phenotype failed, which may affect angiogenesis during the proliferative stage. Secondly, the plasma levels of pigment epithelium-derived factor (PEDF) were found to be elevated in DFU patients and db/db mice. Excessive PEDF reduced the angiogenesis of wound skin, decreased the function and number of endothelial progenitor cells (EPC) in diabetic mice, and delayed wound healing. On the other hand, angiopoietin-1 (Ang 1) and angiopoietin-2 (Ang 2) are important pathways for angiogenesis and maintenance, and in diabetic wounds, Ang 2 continues to be significantly up-regulated and the Ang 2/Ang 1 ratio is dysregulated, which interferes wound angiogenesis (32). Researchers found that the skin of patients with DFU showed up-regulation of CYP1A and SLCO2A1 (24). CYP1A is associated with skin barrier function (33), SLCO2A1 is abundant in vascular endothelial cells (34), and it was found that inhibiting SLCO2A1 can accelerate diabetic wound healing (35).

4. Peripheral neuropathy: Diabetic peripheral neuropathy is caused by many factors, such as oxidative stress, hypoxia, AGEs, activation of T lymphocytes and deficiency of nerve growth factor (NGF). Neuropeptides are neuromodulators that are involved in a variety of processes, including diabetic wound healing. Diabetes mellitus leads to autonomic nerve and small sensory nerve fiber neuropathy, which is characterized by decreased neuropeptide expression (36). The expression of neuropeptide Y in the skin of diabetic patients and diabetic rats decreased. Diabetic neuropathy is a group of heterogeneous diseases with various clinical manifestations. Up to 50% of diabetic peripheral neuropathy (DPN) may be asymptomatic, so early identification and appropriate treatment are necessary, so it is necessary to explore the significance of neuropeptides in diabetic wound healing.

5. Abnormal expression of matrix metalloproteinases (MMPs): When the wound is in the remodeling stage, matrix metalloproteinases decompose collagen, fibronectin and other protein components in the extracellular matrix (ECM), which affects the remodeling of the ECM. MMPs are abnormally active in the skin of patients with diabetic ulcers and are imbalanced with their tissue inhibitors of metalloproteinases (TIMPs). Lobman et al. found that MMP-1, MMP-8, MMP-9 and activated MMP-2 levels were significantly higher in DFU than in normal wounds from non-diabetic patients, while the levels of TIMP-2 were significantly lower than in wounds from non-diabetic patients. Muller et al. reported that high expression of MMP-1 in DFU is critical for wound healing, but excess MMP-8 and MMP-9 may delay wound healing, and the MMP-1/TIMP-1 ratio may reflect the proteolytic environment of the wound (37–39).

6. Abnormal apoptosis: In the process of wound healing, different cell groups are also facing different stages of clearance, until apoptosis. In DFU trauma, mitochondrial damage leads to the up-regulation of pro-apoptotic proteins, while the expression of anti-apoptotic proteins such as B-cell lymphoma -2 (Bcl-2) decreases, leading to apoptosis in various cells such as fibroblasts and vascular smooth muscle cells (**Figure 1**).

Recently, it has been shown that FGF-2 expression is low in wound cells of diabetic patients, and FGF-2 is related to fibroblast mitosis and cell viability. APOD, which is associated with fibroblast regeneration, and CSTB, SMARCA4, and HSPA9 are all expressed at a lower level, which also contributes to the abnormal apoptosis of fibroblasts (40). It has been found that inflammatory cells in diabetic mice show delayed apoptosis during the inflammatory stage of wound healing (41). In the period of proliferation and remodeling, excessive apoptosis of effective cells in high glucose state leads to poor structural recombination, which makes it difficult to generate granulation tissue and makes the wound susceptible to infection (42). Oxidative stress under long-term high glucose condition, accompanied by abnormal glucose and lipid metabolism, leads to long-term chronic inflammation of wounds throughout all stages of wound healing. Patients with DFU disease usually have macroangiopathy, neuropathy and microcirculation abnormalities, involving multiple cells and molecules, which interact with each other very complex, restrict and complement each other.

## FGFs Overview

In 1973, researchers identified the activity of a kind of protein “fibroblast growth factor” from bovine pituitary extract for the first time. This kind of protein stimulated the growth of 3T3 cells at low concentration, which was an established mouse fibroblast cell line (43). It was partially purified in 1975 and purified to homogeneity in 1983 (44), which was called basic fibroblast growth factor (FGF-2 or bFGF) because of its basic amino acid composition and isoelectric point of 9.6 (45). In the same year, it was reported that researchers isolated and purified an active factor without myelin basic protein fragments from bovine brain, and identified that it had the second fibroblast growth factor-like activity, which was called acid fibroblast growth factor (FGF-1 or aFGF) because of its low isoelectric point (46).

The mammalian fibroblast growth factor family contains 22 genes, 18 of which signal through fibroblast growth factor tyrosine kinase receptors, and the other four do not secrete or interact with fibroblast growth factor receptors obviously (47). At present, FGFs are divided into five paracrine FGF subfamilies (i. e. FGF 1, FGF 4, FGF 7, FGF 8, and FGF 9 subfamilies), one endocrine FGF subfamily (i. e. FGF 15/19 subfamily), and one intracellular FGF subfamily (FGF 11 subfamily, also known as iFGFs) based on their biochemical function, sequence similarity, and evolutionary relationship. FGF 15 and FGF 19 may be homologous genes in vertebrates, which are named FGF-15 in rodents but FGF-19 in other vertebrates.

The FGF 1 subfamily consists of FGF-1 and FGF-2. FGF-1 and FGF-2 are present in the nucleus of some cells, and although the mechanism by which FGFs are transported through the cell is unknown, they need to bind to and activate cell surface tyrosine kinase FGFRs, with heparin/HS as a co-factor, and interact with HSP 90 (48). FGF-1 and FGF-2 lack signal peptides, so FGF-1 and FGF-2 are not secreted, but can be released from damaged cells, or through an exocytic mechanism independent of the ER-Golgi pathway (49). It has been suggested that potential functions of FGF 1 include regulating cell cycle, cell differentiation, survival and apoptosis (50). More importantly, FGF-1 is the only one that can activate all FGFR varieties.

According to phylogenetic analysis, the FGF 4 family consists of FGF-4, FGF-5, and FGF-6 (51). In addition, all members of this subfamily have cleavable N-terminal signal peptides, which mediate biological reactions as extracellular proteins by binding fibroblast growth factor receptors and activating IIIc splicing variants of FGFR 1-3 and FGFR 4 (52). The FGF 7 subfamily is composed of FGF-3, FGF-7, FGF-10, and FGF-22, but there is some controversy about the presence of FGF-3 in this family, and the concept of an eighth subfamily composed of FGF 3 only has been proposed (53). The FGF 8 subfamily consists of FGF-8, FGF-17, and FGF-18. Similarly, these FGFs all contain an N-terminal cleaved signal peptide. The FGF 9 subfamily is composed of FGF-9, FGF-16 and FGF-20 although FGF-9 has high secretion efficiency, there is no classical signal peptide at the nitrogen terminal. As early as the early 20th century, it was shown that the hydrophobic region in its structure is key to its secretion and can be transported to the endoplasmic reticulum and secreted from cells as a non-cleaving signal (54, 55).

The FGF 15/19 subfamily consists of FGF-15/19, FGF-21, and FGF-23. Unlike previous FGFs, these FGFs have a very low affinity for heparin/HS binding, so they act mainly as exerting endocrine factors and are known as endocrine FGFs. For endocrine FGFs, they act as cofactors through the Klotho family of proteins, namely αKlotho, βKlotho, and γKlotho (also known as Klotho-LPH-associated protein (KLPH) or lactase-like Klotho (Lctl)), and undergo biological effects after activation and binding to the FGFR receptor, rather than heparin/HS. It is also due to the reduced affinity of FGF 19 subfamily for heparin binding that promotes the release of extracellular matrix (ECM), allowing these FGFs to act as endocrine factors. Among the above FGFs, FGF-1, FGF-2, FGF-4, FGF-7, FGF-21, and FGF-23 had an effect on the treatment of diabetic ulcers (**Table 1**). Additional FGF-11, FGF-12, FGF-13, and FGF-14 are uniformly assigned to intracellular FGFs (iFGFs), also known as the FGF 11 subfamily. IFGFs are neither secreted nor interact with FGFRs, but instead interact with the carboxy terminus of the cytosol of voltage-gated sodium (Nav) channels. Available studies suggest a broad and important role for iFGFs in controlling excitability throughout the central nervous system (62).

**Table 1:** Summary of FGFs related to diabetic ulcer and their characteristics.

Growth Factor	Alternative Symbol	Associated Cofactor	Receptor Specificity	Major effect	Family	Reference
FGF-1	aFGF	HSPGs	All FGFRs	Mitogenic for fibroblast and endothelial cells	FGF1 Subfamily	(56)
				Promotes angiogenesis		
FGF-2	bFGF	HSPGs	FGFR1c,3c,2c,1b,4	Mitogenic for fibroblast and endothelial cells	FGF1 Subfamily	(57)
				Induces cells apoptosis		
FGF-4	kFGF	HSPGs	FGFR1c,2c,3c,4	Stimulates matrix metalloproteinases	FGF4 Subfamily	(58)
FGF-7	KGF	HSPGs	FGFR1b,2b	Mitogenic for keratinocytes	FGF7 Subfamily	(59)
				Promotes epithelialization		
FGF-21		βKlotho	FGFR1c,3c	Reduces inflammation	FGF15/19 Subfamily	(60)
				Promotes re-epithelialization		
FGF-23		αKlotho	FGFR1c,3c,4	Improves vascular calcification	FGF15/19 Subfamily	(61)

FGF, fibroblast growth factor; aFGF, acidic fibroblast growth factor; bFGF, basic fibroblast growth factor; kFGF, Kaposi sarcoma fibroblast growth factor; KGF, keratinocyte growth factor; HSPGs, heparan Sulfate Proteoglycans; FGFR, fibroblast growth factor receptor.

## FGFs-FGFRs Signaling Pathways

FGFs mainly regulate a variety of intracellular responses by binding or activating tyrosine kinase receptors/fibroblast growth factor receptors (FGFRs) on the cell surface (63). There are currently four known FGFRs, namely FGFR1, FGFR2, FGFR3, and FGFR4 (14). Paracrine FGFs and FGFRs bind outside the cell to dimerize FGFRs, and FGFR intracellular tyrosine kinase is activated by auto-trans-phosphorylation.

The activated FGFRs regulate the following signaling pathways in the cell through adaptor proteins (64): 1) Rat sarcoma (RAS)-MAPK pathway: fibroblast growth factor receptor substrate 2α (FRS2α) interacts with Crk-like protein (CRKL) and is phosphorylated by FGFRs kinase. Phosphorylated FRS2α recruits growth factor receptor-bound protein 2 (GRB2), then it recruits guanine nucleotide exchange factor SOS. Furthermore, the recruited SOS activates the RAS GTPase. Finally, the MAPK pathway is activated (65). MAPK can activate the negative regulators CBL, SPRY, SEF, and DUSP6 of the FGF signaling pathway. 2) Phosphatidylinositol 3 kinase (PI3K)-protein kinase B (PKB, AKT): The recruited GRB2 continues to recruit GRB2-associated binding protein 1 (GAB1), then they activate PI3K to phosphorylate AKT (66). 3) Phospholipase Cγ (PLCγ) pathway: activated FGFRs kinase recruits and activates the enzyme PLCγ to produce inositol triphosphate (IP3) and diacyl glycerol (DAG). IP3 induces the release of calcium ions, and DAG activates protein kinase C (PKC) (67). 4) Signal transducers and activators of transcription (STAT) pathway: FGFRs kinase also activates STAT1, STAT3, and STAT5 (68).

**Figure 2:**
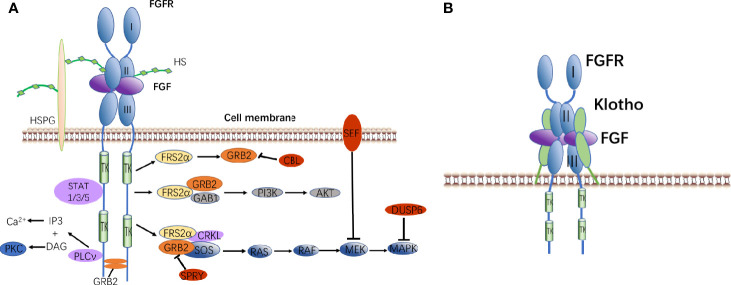
FGF signaling pathways. **(A)** Binding of canonical FGFs to FGFR with HS/HSPG as a cofactor induces the formation of ternary FGF-FGFR-HS complex. The activated receptor is coupled to intracellular signaling pathways including the RAS-MAPK, PI3K-AKT, PLCν, and STAT pathways. **(B)** Binding of endocrine FGF to FGFR with Klotho as a cofactor induces the formation of ternary FGF-FGFR-Klotho complex.

Endocrine FGFs need to rely on Klotho to interact with receptors. The mechanism of action is slightly different from that of paracrine FGFs. FGF-FGFR-Klotho forms a ternary complex that promotes the dimerization of monomeric FGFR. After phosphorylation of FRS2α, it activates signal transduction pathways (**Figure 2**).

## FGF-1 and Diabetic Wound Healing

FGF-1 is called acidic fibroblast growth factor (aFGF), which mainly distributes in organs or tissues, such as heart, brain, adrenal gland, pituitary gland, nerve tissue, retina and bone. Since its isolation and purification in 1984, it has been confirmed that aFGF has the effects of promoting injury repair, promoting angiogenesis, protecting and nourishing neurons. As early as 1994, it was found that acidic fibroblast cytokines can accelerate skin wound healing in diabetic mice (69). By comparing the skin growth factors of diabetic animals with those of non-diabetic animals, and observing the changes in the expression and content of the corresponding genes, the researchers found that the gene expression and content of aFGF and bFGF were indeed decreased in the early stage of diabetic wound healing, and the gene expression of aFGF and bFGF was advanced (70). A low dose FGF-1 injected intraventricular into diabetic mouse model can produce sustained hypoglycemic effects, and its advantage is that this effect will not increase the risk of hypoglycemia (71). The researchers measured serum FGF-1 levels in patients with T2DM by ELISA for the first time, and the results showed that body mass index and glycosylated hemoglobin were independent factors affecting serum FGF-1 levels, serum FGF-1 levels were significantly associated with T2DM, and FGF-1 blood concentrations were significantly increased in diabetic patients (72).

Various studies have shown that FGF-1 is closely related to the healing of diabetic wounds. Therefore, the effects of FGF-1 on wound healing were summarized by referring to the literature: 1. Improving cell proliferation ability: FGF-1, as a mitogen, can promote the mitosis of mesoderm and ectoderm-derived cells, promote the proliferation of epidermal epithelial cells, and contribute to the epithelialization of wounds (73). It stimulates the proliferation and migration of fibroblasts and keratinocytes, causes the migration of inflammatory cells and wound edge cells to the wound surface, and induces the production of proteases, collagenases, and various cytokines (74). 2. Promoting synthesis of extracellular matrix: FGF-1 can regulate the proliferation and differentiation of collagen, collagen fibers and fibroblasts in granulation tissue. Then a new extracellular matrix was synthesized to increase collagen content and stimulate fibroblasts and endothelial cells to secrete collagenase and proteolytic enzymes. Finally, plasminogen activator breaks down collagen, and the synthesis and breakdown of collagen fibers can balance the collagen content in the wound tissue (75). 3. Promote angiogenesis, proliferation and differentiation: FGF-1 is also an extremely potent angiogenic factor, and its pro-angiogenic effect has long been confirmed, promoting the formation of new capillaries, increasing the blood supply of the wound, accelerating granulation growth, and making tissue repair and epidermal regeneration (76). It has been shown that FGF-1 can protect blood vessels from oxidative stress *in vitro* and *in vivo* (77). Angiogenesis is mainly mediated by angiogenin and growth factors. FGF-1 enhances the synthesis of angiogenin and then promotes angiogenesis (56). FGF-1 can significantly increase the number of capillaries and fibroblasts in ulcer tissue, and enhance the expression of transforming growth factor-β and nuclear antigen proliferating protein (PCNA), thus improving diabetic ulcer tissue (16).

At present, the development of FGF-1 as a new clinical drug for the treatment of diabetic ulcer healing is advancing constantly. NONcNZO10/LTJ mice is a new multi-gene strain, which can simulate human metabolic syndrome and obesity-induced type 2 diabetes more realistically. The results of NONcNZO10/LTJ mouse studies enhance the potential of aFGF in treating skin wounds in diabetic patients, because researchers found that when exogenous heparin is not added in the formula, stable mutant aFGF may obtain similar therapeutic effects, but it has greater potential safety and cost advantages than wild-type aFGF proteins, and the reconstruction of fibroblast growth factor -1 (aFGF) is identified as a potential “second generation” therapy to promote the healing of skin wounds of diabetes (78). In order to improve the application limitation of low administration efficiency and short half-life, transcription protein transactivator (TAT) -aFGF was used for treatment. The results showed that TAT-aFGF had a good therapeutic effect on deep subcutaneous tissue injury healing (79). Another study showed that endothelial progenitor cell-derived exosomes (EPC-Exos) transplantation can promote the healing of skin wounds in diabetic rats, and thus promote the proliferation, migration and tubular formation of vascular endothelial cells *in vitro*. The results showed that EPC-Exos could significantly up-regulate the expression of various key pro-angiogenic genes such as aFGF by more than 4-fold and actively regulate the function of vascular endothelial cells to promote wound healing (80).

Various studies have shown that FGF-1 can enhance diabetic wound healing and has shown its great potential as a treatment for diabetic ulcers. Now, researchers should continue to conduct in-depth research to improve the application limitations of FGF-1, so that FGF-1 can be used clinically as soon as possible.

## FGF-2 and Diabetic Wound Healing

FGF-2 is also called basic fibroblast growth factor (bFGF). The expression of bFGF in angiogenesis, neurogenesis and neuron survival. Many previous studies have shown that the difficulty in healing diabetic wounds may be related to local hyperglycemia and accumulation of advanced glycation end products (AGEs) in diabetic skin (81). On the other hand, glycosylated bFGF inhibits the proliferation and angiogenesis of human skin microvascular endothelial cells (HDMEC) through the RAGE pathway. Further experiments show that glycosylated basic fibroblast growth factor shows more negative effects in the process of wound healing, which may be one of the reasons for slow healing of diabetic wounds (82).

Since 1990, studies have shown that treating incisions of diabetic rat with bFGF can improve epithelialization, granulation and wound tear strength to levels of non-diabetic mice (83), and clinical patients have also shown that bFGF can indeed accelerate the healing of diabetic ulcers (84). bFGF has mitogenic properties (85), and accelerate the division and proliferation of endothelial cells, skin fibroblasts and keratinocytes (86). In turn, it affects the migration of these cells during wound healing, thus promoting the formation of new blood vessels and epithelia. Studies have shown that controlled release of bFGF accelerates the healing of skin wounds and increases the epithelial formation rate in a diabetic mouse. The angiogenic and mitogenic effects of bFGF significantly stimulate the proliferation period of wound healing, and induce apoptosis of fibroblasts and myofibroblasts in wound, thus reducing scar formation during healing (17). *In vitro* and *in vivo* studies have shown that bFGF promotes dermal fibroblast migration in diabetic patients by independently activating the PI 3K/Akt-Rac 1-JNK pathway to increase ROS production (57, 87). It has been shown that the expression level of FGF-2 is decreased in wounds of diabetic animals, which is related to the significant delay of angiogenesis, fibrous hyperplasia, and collagen formation. In the later stages of DFU healing, this delay is manifested by reducing angiogenesis, fibrous proliferation, and collagen, which is associated with reduced expression of FGF-2 and sustained expression of TNF-α (88).

As early as 1995, researchers treated patients with diabetic nerve foot ulcers with topical FGF-2 treatment, but the treatment results were not satisfactory. The results of the study found that topical FGF-2 treatment has less improvement in the healing of diabetic ulcers, and may require a combination of several topical applications and special growth factors. There is another reason that cannot be ignored. FGF-2 is locally degraded and/or absorbed into excipients, thereby losing its efficacy. Adding FGF-2 to gels or creams may have a significant impact (89). Subsequently, it has been shown that FGF-2 retained in chitosan hydrogels is biologically active, from which FGF-2 molecules are released when the hydrogels are biodegraded *in vivo*. In db/db mice, wounds treated with FGF-2 incorporated into chitosan hydrogel showed significant granulation tissue formation, capillary formation and epithelial formation (90). In recent years, an optimized wound healing gel could provide proteoglycan-4 and FGF-2 to promote diabetic wound healing. *In vitro* studies showed that the complex significantly increased the migration of keratinocytes and fibroblasts. In addition, combined treatment increased the endocytosis process of FGF-2, including enhanced recycling of FGF-2 to the cell surface after ingestion (91). Secretory neuropeptide SN is a kind of angiogenic neuropeptide. *In vitro* experiments show that the interaction between FGF-2 and secreted neuropeptides can play an active role in angiogenesis, proliferation and apoptosis of cells, and FGFR 3 is the main receptor mediated by secreted neuropeptide. The investigators hypothesize that FGF-2 with local SN gene therapy may treat microvascular dysfunction in DFU patients (92). Through in-depth study of FGF-2 structure, the researchers synthesized homodimers of FGF-2 linked at both ends of polyethylene glycol, and FGF-2-PEG-2k-FGF-2 showed better activity in the metabolic activity and migration of human umbilical vein endothelial cells compared with FGF-2, and promoted angiogenesis in an *in vitro* co-culture model. FGF-2-PEG-2k-FGF-2 increased wound granulation tissue and vascular density, as assessed in an *in vivo* wound healing model in diabetic mice (93). In another study on the structural modification of FGF-2, the researchers developed a more stable basic fibroblast growth factor (ST-bFGF) to overcome the limitations of commercial FGF-2 (CA-bFGF), which has a short half-life and loses its activity after being loaded into the matrix (94). In recent years, it has become the mainstream to search for potential drugs for treating delayed healing of diabetic wounds, and FGF-2 has also become a condition for its evaluation. For example: lupeol, a triterpenoid compound, is found in many medicinal plants. Treatment of diabetic skin wounds with lupeol showed that inflammatory cell infiltration decreased, fibroblast proliferation, angiogenesis and collagen fiber deposition increased, which led to the increased expression of FGF-2. Therefore, the evaluation of lupeol may have therapeutic effects on chronic wounds in diabetic patients (95). In addition, topical application of a combination of hydroalcoholic extract of ryegrass (TPEO) and aloe vera gel (AVGO) significantly increased FGF-2, shortened the inflammatory phase, and increased cell proliferation and collagen deposition, thereby accelerating the healing of diabetic open wounds (96).

FGF-2 is one of the most widely studied FGFs. In recent years, several studies have shown that FGF-2 does have a positive effect on the healing of diabetic ulcers. Therefore, further optimization of FGF-2 excipients to maximize the therapeutic effect of FGF-2 has become a more concerned issue.

## FGF-4 and Wound Healing

FGF-4 is also known as Kaposi’s sarcoma FGF. In adults, FGF-4 mainly exists in tumors, such as gastric cancer, Kaposi’s sarcoma and breast cancer, and is not produced under normal physiological conditions. FGF-4 is the first fibroblast growth factor defined as an oncogene, which is expressed throughout the whole embryonic development (97). However, it has been found that FGF-4 stimulates the expression of matrix metalloproteinase -9 (MMP-9) and VEGF receptor-1 in mouse skin fibroblasts *in vitro*, and its combination with VEGF-A promotes the migration of fibroblasts and accelerates wound healing in diabetic mice (58). The findings provide a new idea for the treatment of diabetic ulcers, but whether it has important clinical significance remains to be determined, which deserves further preclinical and clinical research.

## FGF-7 and Diabetic Wound Healing

Keratinocyte growth factor KGF-1 is the seventh structurally related member of the fibroblast growth factors (FGFs) family and is named FGF-7. As a paracrine growth factor, it is not produced by keratinocytes but by various cells (i.e., fibroblasts, ECs, smooth muscle cells, and dendritic epidermal T cells). Fibroblast-treated wounds may stimulate keratinocyte proliferation and accelerate healing through re-epithelialization. Re-epithelialization depends on the proliferation and migration of keratinocytes at the wound edge. In fibroblast-treated wounds, both microscopic keratinocytes in an active proliferation state and macroscopic re-epithelialization were stimulated, which is consistent with a significant increase in the level of FGF-7 in the wound (98). FGF-7 has been shown to stimulate the migration and proliferation of keratinocytes (59, 99).

Since the early 20th century, it has been proved that although FGF-7 deficiency does not seem to affect re-epithelialization of skin wounds in diabetic mice, it can significantly reduce the shrinkage rate of wound healing by further changing the skin composition of diabetic mice. Due to the specific targeting of FGF-7 to epithelial cells, there is further epithelial-intercellular interaction dependent on FGF-7, which may play an important role in diabetic wound healing (100). However, another study showed that FGF-7 is a growth factor and a chemical inducer of fibroblasts. As one of the main components of the epithelium is keratinocytes, FGF-7 accelerates the proliferation of keratinocyte. The formation of granulation tissue, the promotion of angiogenesis and epithelization are important treatment methods for chronic wound healing (101). In addition, new research shows that cells obtained in dermal connective tissue can be used as mesenchymal stem cells, because the reduction and delay of FGF-7 growth factor production in diabetic patients improves with the improvement of this cell transplantation, and contribute to wound healing (102). FGF-7 is damaged in diabetic skin wounds, but the specific regeneration mechanism remains to be proved. The effect of FGF-7 on foot injury of diabetic patients and its regeneration mechanism needs to be explored, but this provides a reference for DFU patients with slow wound healing.

## FGF-16 and Diabetic Wound Healing

In recent years, researchers have continuously explored and found that miR-144-3 p inhibits high glucose-induced cell proliferation by inhibiting FGF-16 and MAPK signaling pathways, suggesting that miR-144-FGF-16 may play a role in diabetic wound healing (103). However, a large number of studies and experiments are needed to confirm how miR-144-FGF-16 inhibits the deeper mechanism of high glucose cells and explore whether it can be used as a new target for the treatment of DFU.

## FGF-21 and Diabetic Wound Healing

FGF-21, a member of the FGF 15/19 subfamily, is mainly expressed in the liver and adipose tissue, secreted in skeletal muscle, myocardium, pancreas and hypothalamus, and is an endocrine factor with various functions. According to the literature records, FGF-21 can regulate glucose and lipid metabolism, maintain energy balance, and play a role in regulating blood lipid and resisting oxidation (104), protect the blood-brain barrier from traumatic brain injury and prevent the blood-brain barrier leakage in type 2 diabetes mellitus (105, 106), and promote remyelination and functional recovery of injured peripheral nerves. FGF-21 plays an important role in the treatment of type 2 diabetes, while FGF-21 has the advantage of no obvious side effects, such as edema and hypoglycemia (107).

The main reasons for delayed ulcer healing caused by diabetes are long-term exposure to high glucose, over-expression of oxidative stress and inflammatory reaction. It has been reported that FGF-21 can reduce the oxidative stress and inflammation of photoreceptors through metabolic regulation, thereby improving the function of photoreceptors (60). A series of experiments on diabetic mouse models showed that recombinant FGF-21 had biological activity, which may promote skin wound healing, early granulation tissue formation, uniform collagen deposition and re-epithelialization by activating the ERK signaling pathway or inhibiting apoptosis. In the middle and later stages of wound healing, it was also confirmed that a large amount of collagen type I and type III were deposited in the wound, which indicated that the formation of new blood vessels was promoted (108). It has been proved that FGF-21 can directly inhibit the proliferation and migration of vascular smooth muscle cells in diabetic mice, leading to restenosis after injury of vascular smooth muscle cell in diabetic mice. In addition, it was found that FGF-21 inhibited the formation of NLRP 3 inflammatory body in vascular smooth muscle cells under hyperglycemia stress through FGFR 1, and inhibit the formation of NLRP 3 inflammatory body in vascular smooth muscle cells by inhibiting the dephosphorylation of Syk tyrosine 525 (109). FGF-21 as also been studied to regulate wound inflammatory response, and FGF-21 can reduce excessive inflammatory response in rat skin and promote the transformation of inflammatory phase into proliferative phase by up-regulating IL-10, down-regulating IL-6, IL-1β and TNF-α (110).

## FGF-23 and Diabetic Wound Healing

FGF-23 is a new secretory protein produced by bone cells and osteoblasts, which has been proved to be an important regulator of calcium and phosphorus metabolism in human (61). Recent clinical studies have shown that the increase of serum FGF-23 levels is an independent risk factor for lower limb artery disease in Chinese diabetic patients. Studies have reported decreased serum Klotho concentrations and increased FGF-23 in patients with diabetic foot, and importantly, these parameters are independently associated with diabetic foot ulcers. These results indicate that the lower the risk of diabetic foot ulcer, the lower the concentration of serum Klotho and the expression level of vascular genes, and the lower the risk of diabetic foot syndrome. These findings indicate a new pathophysiological pathway that may be related to the delayed healing of diabetic wounds, but further research is needed to clarify the role of the FGF-23/Klotho system in the development and progression of this complication (111).

## Current Surgical Treatment of DFU

DFU can lead to the development of chronic wounds, which usually leads to amputation. Effective and timely treatment intervention can control wound infection, promote healing and reduce the amputation rate. Up to now, various classifications have been used to evaluate the severity of diabetic foot diseases. The most commonly used classification is Wagner’s 6-grade classification: (1) Grade 0: It is a high-risk foot with no obvious ulcer in the clinic. (2) Grade 1: Superficial ulcer without exposed tendon joints (3) Grade 2: Relatively deep penetrating ulcers, often accompanied by cellulitis, without abscess or bone infection, and special bacteria exist in the ulcer site. (4) Grade 3: Deep skin ulceration, often involving bone tissue, accompanied by abscess or osteomyelitis. (5) Grade 4: Ischemic ulcer, local or digital gangrene, and gangrene without pain indicate neuropathy. (6) Grade 5, most or all of the feet are gangrenous. The characteristics of the ulcer are well described to make a treatment plan.

At this stage, local adjuvant therapy is usually used for wound treatment of DFU. There are several methods of local auxiliary treatment: 1. Antibiotics can be used alone or in combination to minimize drug resistance and adverse reactions. 2. Hyperbaric oxygen shows good therapeutic prospects in the treatment of severe nonunion DFU disease. Hyperbaric oxygen therapy has been shown to sensitize macrophages and release FGF-2 and epidermal growth factor, thus promoting angiogenesis. Hyperbaric oxygen therapy promotes wound healing by increasing oxygen supply and distribution to damaged tissues, stimulating angiogenesis, reducing inflammation and increasing nitrite levels. The elevation of Nrf 2 level can temporarily regulate the expression of angiogenesis genes in wound biopsies, which may lead to accelerated healing of chronic wounds (112). 3.Electrical stimulation therapy is an effective adjunctive therapy for diabetic foot ulcers (113), but more experimental and clinical studies are needed to elucidate its mechanism affecting chronic wound regeneration. 4. For a long time, debridement has been proved to be the most important treatment step to close the diabetic foot wound and reduce the amputation rate (114). 5. Selection of appropriate excipients that can promote wound healing, such as the addition of silver ions to hydrogel dressings has potential in the treatment of diabetic foot ulcers (115). Our previous research showed that asiaticoside NO gel may promote diabetic cutaneous ulcers wound healing by regulating Wnt/β-Catenin signaling pathway (116). Our next goal is to continue to study more suitable dressing according to the etiology of DFU, location, depth and exudate of the wound bed. 6. Tissue engineered skin substitutes are widely used in the treatment of chronic wound healing (117). However, the application of tissue-engineered skin is still limited due to peripheral ischemia. 7. Negative pressure wound therapy (NPWT) has been successfully applied to DFU (118). 8. Biotherapy: FGF helps patients with type 1 and type 2 diabetes to heal wounds caused by DFU through parenteral evaluation applied to ulcer surface (alone or loaded in dressing matrix) or directly penetrating into wounds (94, 119). 9. Surgical treatment: According to the condition of the wound and the patient’s physical condition, debridement or skin grafting can effectively remove necrotic tissue.

## Current Research Progress and Limitations of FGFs in the Treatment of DFU

**Figure 3:**
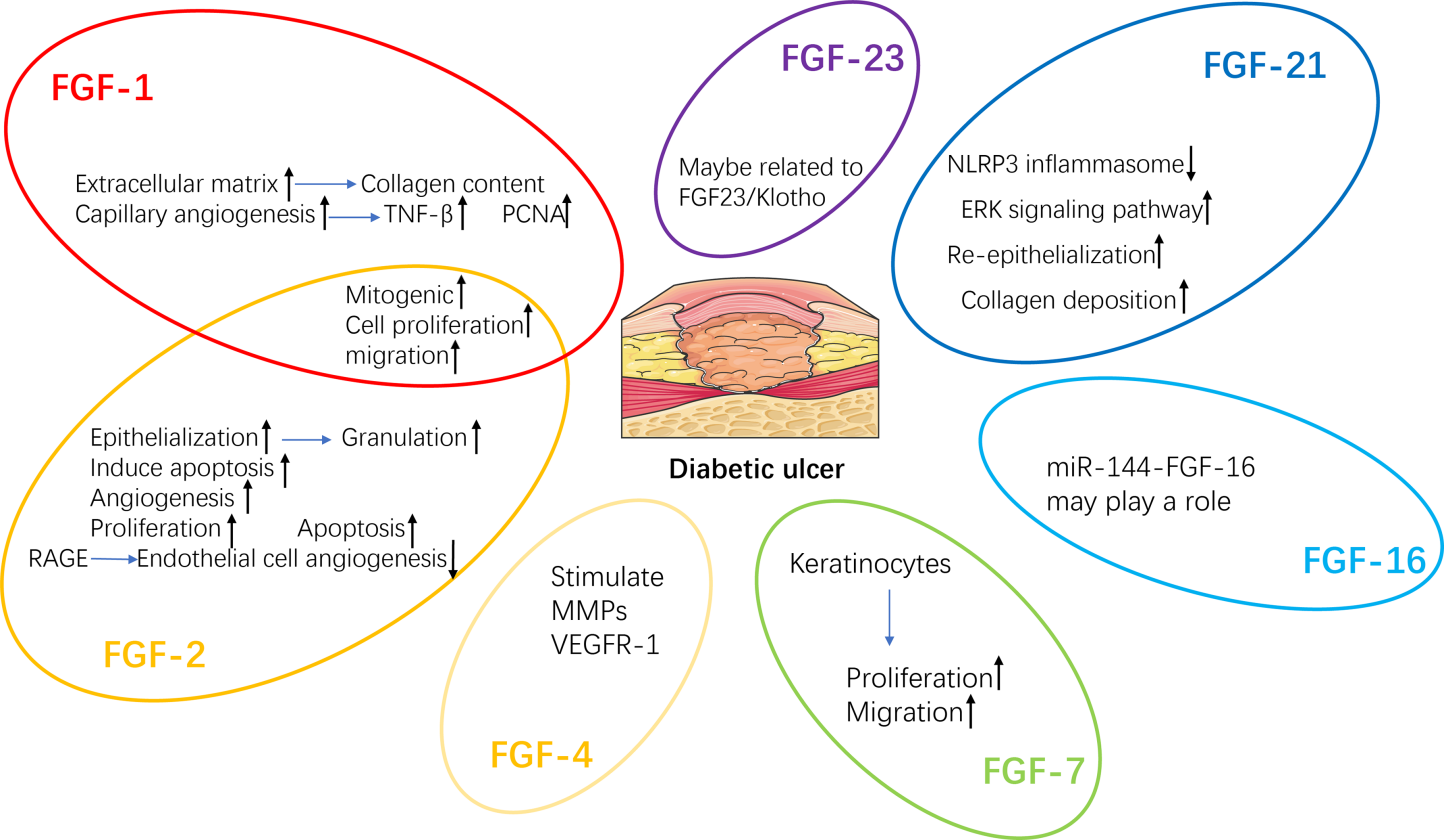
Regeneration mechanisms of FGFs related to diabetic wound healing. TNF-β, Tumor necrosis factor-β; PCNA, Proliferating cell nuclear antigen; VEGFR-1, Vascular endothelial growth factor receptor-1; MMPs, Matrix metalloproteinases.

Delayed wound healing is one of the main reasons for lower limb amputation in diabetic patients (120). A variety of growth factors, such as fibroblast growth factor (FGF) (**Figure 3**), platelet-derived growth factor (PDGF), vascular endothelial growth factor (VEGF) and insulin-like growth factor (IGF), change in patients with DFU. Therefore, the healing process of diabetic wounds can be accelerated by regulating the level of growth factor (15).

Previous experiments showed that 48% of 61 patients treated with recombinant human platelet-derived growth factor PDGF-BB were completely healed, while 25% of 57 patients treated with placebo were completely healed (121). PDGF is currently approved by the US Food and Drug Administration (FDA) for the treatment of diabetic neuropathic ulcers of the lower extremities (122, 123). In 2000, the American Food and Drug Administration of China approved the use of recombinant human basic fibroblast growth factor (rb-FGF 2) to treat chronic wounds, including ulcers, bedsores and burns. The researchers reported the application of FGF 2 eye drops in the treatment of mechanical superficial corneal abrasions (124); Subsequently, the researchers also observed the therapeutic effect of Rb-FGF 2 on the feet of early diabetic patients. In 2005, recombinant human acidic fibroblast growth factor (rh-FGF 1) was developed by Chinese scientists and approved by the China Food and Drug Administration as the first marketed FGF-1 drug in the world (125). In 2006, rh-FGF 1 was marketed for the treatment of diabetic ulcers (126).

However, FGFs have some limitations for the treatment of DFU wound healing, since growth factors generally have a short half-life, require repeated administration, and chronic wounds have a high and constant proteolytic environment, these growth factors can be easily degraded.

## Future Research Directions of FGFs

In general, experiments have shown that multiple FGFs mentioned above are closely related to diabetic wound healing. However, the limitations of FGFs have largely hindered the application of FGFs. In order for FGFs to be a potential choice for the treatment of DFUs, improved carriers or delivery methods, such as novel dressings, must be developed, as well as the combination of skin substitutes manufactured by tissue engineering with FGFs for DFUs (127). In addition, whether the long-term single use of FGFs to treat diabetic ulcers produces other systemic side effects remains to be further studied.

## Author Contributions

YeL responsible for literature review and writing. YQL, JD, and WL responsible for correction. XN responsible for proofreading, literature review, and correction. All authors contributed to the article and approved the submitted version.

## Funding

This work was supported by the National Natural Science Foundation of China (81960741, 82160770, 81560712), the Guizhou Provincial Natural Science Foundation (QKH-J-2020-1Z070), the Special Funding for Postdoctoral Research Projects in Chongqing (Xm2019061), Guizhou Provincial Administration of Traditional Chinese Medicine Funding (QZYY2017-080).

## Conflict of Interest

The authors declare that the research was conducted in the absence of any commercial or financial relationships that could be construed as a potential conflict of interest.

## Publisher’s Note

All claims expressed in this article are solely those of the authors and do not necessarily represent those of their affiliated organizations, or those of the publisher, the editors and the reviewers. Any product that may be evaluated in this article, or claim that may be made by its manufacturer, is not guaranteed or endorsed by the publisher.
